# Multi-mtDNA Variants May Be a Factor Contributing to Mitochondrial Function Variety in the Skin-Derived Fibroblasts of Leber's Hereditary Optic Neuropathy Patients

**DOI:** 10.3389/fnmol.2022.920221

**Published:** 2022-07-13

**Authors:** Shun Yao, Qingru Zhou, Mingzhu Yang, Ya Li, Xiuxiu Jin, Qingge Guo, Lin Yang, Fangyuan Qin, Bo Lei

**Affiliations:** ^1^Henan Provincial People's Hospital, Zhengzhou University People's Hospital, Zhengzhou, China; ^2^Henan Eye Institute, Henan Eye Hospital, Henan Provincial People's Hospital, Zhengzhou, China; ^3^Academy of Medical Sciences, Zhengzhou University, Zhengzhou, China

**Keywords:** Leber's hereditary optic neuropathy (LHON), mitochondria dysfunction, heterogeneity, mtDNA variants, cell viability

## Abstract

Heterogeneity is a major feature of Leber's hereditary optic neuropathy (LHON) and has a significant impact on the manifestation and diagnosis of the disease. This study explored whether multiple variations in mitochondrial genes were associated with the heterogeneity, mainly phenotypic heterogeneity. Ophthalmic examinations were conducted in two probands with LHON with G11778A and multiple mitochondrial DNA gene (mtDNA) variants. Skin fibroblast cell lines were generated from patients and age- and sex-matched controls. ROS levels, mitochondrial membrane potential, cell energy respiration, and metabolic functions were measured. Flow cytometry and cell viability tests were performed to evaluate the cell apoptosis levels and fate. We found that cells with more mtDNA variants had higher ROS levels, lower mitochondrial membrane potential, and weaker respiratory function. Flow cytometry and cell viability testing showed that multiple mtDNA variants are associated with different levels of cell viability and apoptosis. In conclusion, we found that skin-derived fibroblast cells from G11778A LHON patients could be used as models for LHON research. Multi-mtDNA variants contribute to mitochondrial function variety, which may be associated with heterogeneity in patients with LHON.

## Introduction

Leber's hereditary optic neuropathy (LHON) is the most common hereditary optic neuropathy with an average onset around age 20. Patients usually suffer from painless, subacute, or rapid vision loss in both eyes (Yu-Wai-Man and Chinnery, 1993; Yu-Wai-Man et al., 2009; Amore et al., 2021; Hage and Vignal-Clermont, 2021). LHON is caused by mitochondrial gene variations, and more than 30 pathogenic mutations have been found (Brandon et al., 2005). Among these variations, *MT-ND4* 11778G>A, *MT-ND6* 14484T>C, and *MT-ND1* 3460G>A account for the majority of LHON patients (Wallace et al., 1988; Liang et al., 2014; Ji et al., 2016; Newman et al., 2020). These mutations cause mitochondria dysfunction, resulting in retinal ganglion cells atrophy and death.

Leber's hereditary optic neuropathy features with significant genetic and phenotype heterogeneity, and manifests in incomplete dominance and male bias (Riordan-Eva et al., 1995; Chalmers and Schapira, 1999; Cui et al., 2020). The severity, age of onset, and penetrance of optic neuropathy vary among maternal relatives even within a family, although same LHON-related variant is carried. It is of great significance to clarify the genetic and phenotype heterogeneity in this disease which may provide a detailed understanding of diagnosis, prognosis, and proposed treatment.

Genetically, in addition to the pathogenic mitochondrial DNA gene (mtDNA) mutations, nuclear gene mutations and epigenetic inheritance are closely related to the occurrence and development of LHON (Bu and Rotter, 1991; Wiggs, 2021). Jiang P et al. found that the combination of non-classical mutation of nuclear gene *YARS2* and mitochondrial gene *MT-ND4* 11778G>A variation aggravated mitochondrial damage, leading to exacerbated phenotype (Jiang et al., 2016; Jin et al., 2021). Besides, nuclear gene *PRICKLE3* and *MT-ND4* mutation also intensify optic nerve injury in a similar synergistic manner (Yu et al., 2020). These studies demonstrated that multi variants presenting in the nuclear and the mitochondrial genes are associated with disease severity, which contribute to the disease heterogeneity.

In addition to the additional mutations in the nuclear gene, multiple variants in the mitochondrial genes may be also associated with the disease heterogeneity. Mitochondrial genes encoding proteins such as MT-ND, MT-CO, and MT-ATP are subunits of the mitochondrial respiratory chain. Variations in these genes directly affect respiratory function and consequently leading to mitochondria dysfunction (Stenton et al., 2021). In this study, we reported two LHON probands with different clinical manifestations, while both cases harbored the *MT-ND4* 11778G>A mutation, one had additional LHON-associated variants. We showed in the patients' skin-derived fibroblast cells that different levels of mitochondrial dysfunction and vulnerability to adverse stimulus, suggesting the number of mtDNA variants may be a factor that contribute to the heterogeneity of LHON.

## Results

### Clinical Features of Mild and Severe Patients

**Figure 1:**
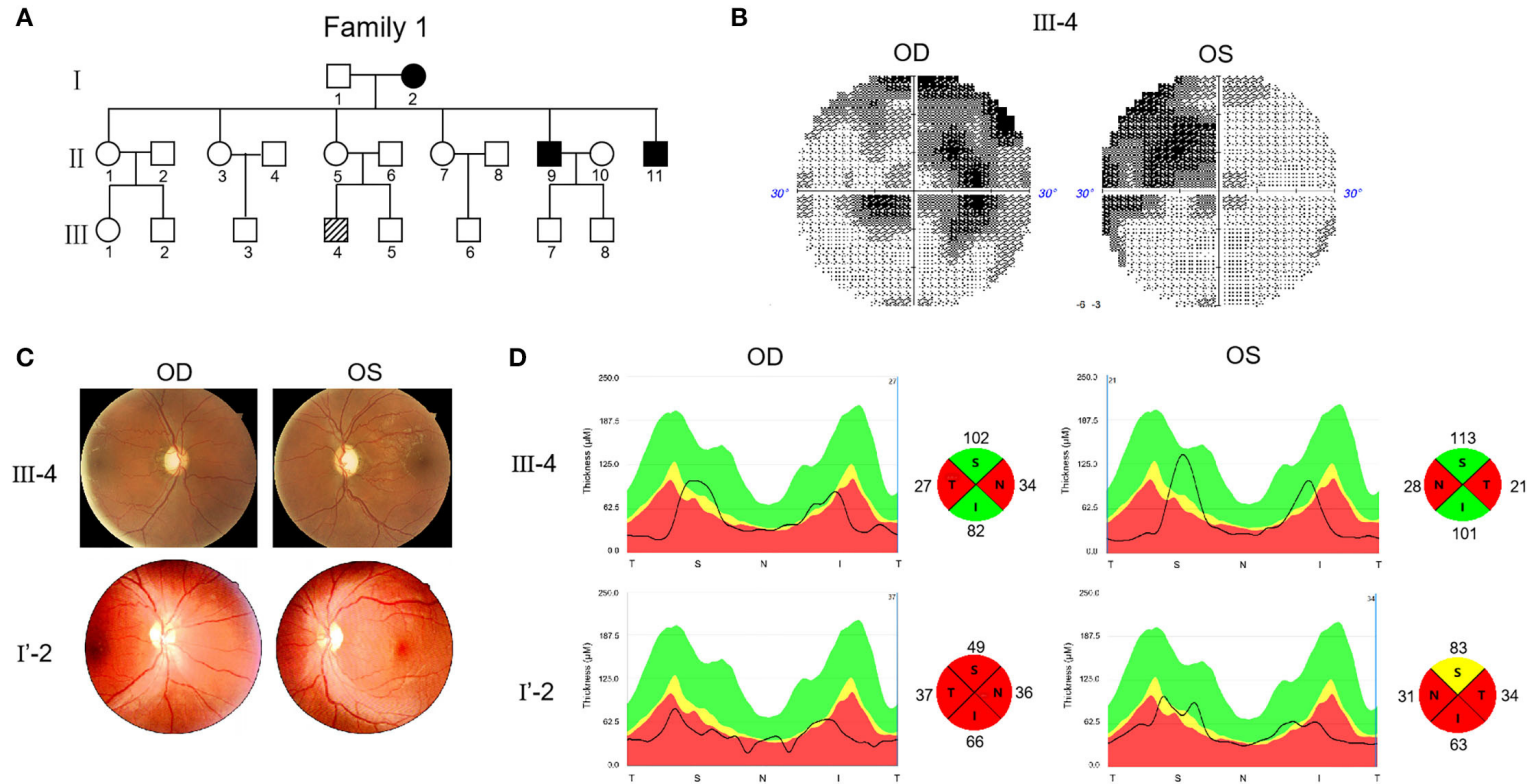
Pedigree and features of probands with Leber's hereditary optic neuropathy (LHON). **(A)** Pedigree of two probands. Arrow denotes the proband. **(B)** Visual field test results of proband III-4. **(C)** Fundus photographs of two probands. **(D)** RNFL assay of two probands after swept-source optical coherence tomography (SS-OCT) detection.

Two Chinese families were included in this study. In Family 1, the proband (III-4) was 28 years old and experienced progressive visual impairment without other symptoms at the age of 12 years (Figure 1A). In 2019, the patient visited the Henan Eye Hospital, and their bilateral BCVA was found to be 0.15. In the next 2 years of follow up, there was no further decrease in vision. Visual field testing demonstrated the nasal scotomata in both eyes (Figure 1B). Fundus photography showed bilateral pale optic disks and a thinner retinal nerve fiber layer (Figure 1C). Swept-source optical coherence tomography (SS-OCT) showed that thickness of the retinal nerve fiber layer in the nasal and temporal regions was attenuated (Figure 1D).

The other proband (I'-2) was 32 years old. No genetic information about their ancestry was available as the patient was adopted. The first visit to the Henan Eye Hospital was in 2019, when the patient complained of progressively loss of vision starting 1 year ago without other symptoms. BCVA was 0.1 in the left eye and 0.15 in the right eye. In the 2 years of follow up, the patient's vision had progressively decreased. In March, 2021, the vision reduced to FC/10 cm in both eyes. Fundus examination revealed pathological optic disks with incomplete boundaries (Figure 1C). SS-OCT revealed severe atrophy of the retinal nerve fibers in both eyes, particularly on the nasal and temporal sides (Figure 1D).

Considering these clinical symptoms, genetic test results and no history of other diseases, we diagnosed LHON in both patients. Both cases were of the same sex, comparable age, and similar baseline BCVA and duration of follow-up. However, the severity and progression of the disease varied significantly. The visual acuity of patient F1 III-4 was 0.15 when he first came to the hospital and there was no further decline after 2 years. This patient was diagnosed with mild LHON. The vision of patient F2 I'-2 dropped from 0.15 to FC/10 cm in 2 years. This patient was diagnosed with severe LHON. SS-OCT further demonstrated that III-4 had optic fiber atrophy only in the nasal and temporal regions, whereas I'-2 showed atrophy in four quadrants.

### The Number and Conservation of Mitochondrial Nucleotide Variants Were Different in Patients With Mild or Severe Symptom

**Figure 2:**
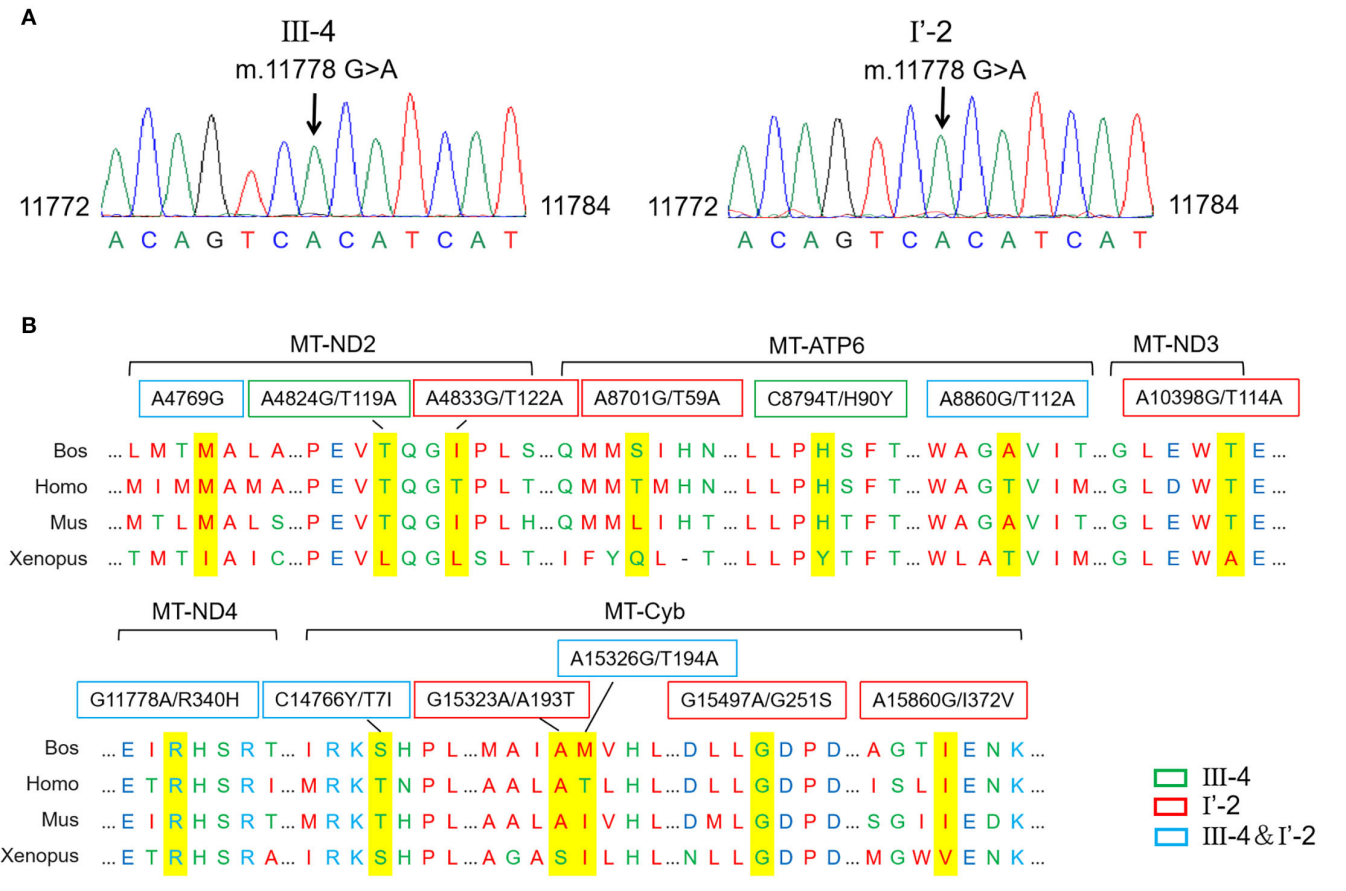
Different mitochondrial DNA gene (mtDNA) variations of two probands. **(A)** The mutation m.11778G>A was confirmed in probands by the Sanger sequencing. **(B)** Conservative analysis of mutant amino acids in four species. Bos, Bostaurus; Homo, Homo sapiens; Mus, Musmusculus; Xenopus, Xenopuslaevis.

An mtDNA 11778G>A pathogenic mutation was found in both probands (Figure 2A). In addition, whole mtDNA sequencing revealed multiple mtDNA polymorphisms: the mild proband (III-4) had 29 variants (Table 1), and the severe proband (I'-2) had 40 variants (Table 2). These variants covered almost all mtDNAs, such as intergenic regions, non-coding regions (12s rRNA and 16s rRNA), and coding regions (*MT-ND, MT-CO, MT-ATP*, and *MT-Cyb*). These variants included missense and synonymy. In proband III-4, six missense variants were present in the coding regions of mtDNA: *MT-ND2* 4824A>G, *MT-ATP6* 8794C>T, *MT-ATP6* 8860A>G, *MT-ND4* 11778G>A, *MT-Cyb*14766C>T, and *MT-Cyb*15326A>G. Proband I'-2 had 11 mistranslation variants in mtDNA, almost twice as many as III-4. These variant loci were *MT-ND2* 4833A>G, *MT-ATP6* 8701A>G, *MT-ATP6* 8860A>G, *MT-ND3* 10398A>G, *MT-ND4* 11778G>A, *MT-Cyb* 14766C>T, *MT-Cyb* 15323G>A, *MT-Cyb* 15326A>G, *MT-Cyb* 15497G>A, *MT-Cyb* 15860A>G, and *MT-Cyb* 15968T>C. A phylogenetic analysis was performed to evaluate the conservation of these variants and sequences from other organisms such as mice, bovine, and *Xenopus laevis*. Among the six missense variants in patient III-4, two variants were conserved in mammals, except for G11778A. Four of the 11 variants in addition to G11778A were conservative in patient I'-2, particularly *MT-Cyb* 15497G>A (Figure 2B).

**Table 1:** The mitochondrial DNA gene (mtDNA) variants in the mild patient (III-4).

**Gene**	**Position^**#**^**	**Replacement (base)^**#**^**	**Gene region^**#**^**	**Mitomap** ** frequency**	**Replacement (amino)^**#**^**	Conservation (H/B/M/X)^*^	**Pathogenicity prediction** ** (APOGEE)**	**References**
	73	A>G	Intergenic					
	152	T>C						
	235	A>G						
	263	A>G						
	309	C>T						
	310	T>C						
12S rRNA	663	A>G	Non-coding					
	750	A>G						
	1438	A>G						
16S rRNA	1736	A>G	Non-coding					
	2706	A>G						
	3106	CN>C						
MT-ND1	4248	T>C	Synonymous					
MT-ND2	4769	A>G	Synonymous					
	4824	A>G	Missense	2.925%	T119A	T/T/T/L	Neutral (score: 0.4)	0.998
MT-CO1	7028	C>T	Synonymous					
MT-ATP8	8563	A>G	Synonymous					
MT-ATP6	8794	C>T	Missense	2.824%	H90Y	H/H/H/Y	Neutral (score: 0.44)	0.998
	8860	A>G	Missense	98.503%	T112A	T/A/A/T	Neutral (score: 0.27)	0.997
MT-ND4	11536	C>T	Synonymous					
	11719	G>A	Synonymous					
	**11778**	**G>A**	**Missense**	**0.341%**	**R340H**	**R/R/R/R**	**Pathogenic (score: 0.97)**	0.997
MT-ND5	12705	C>T	Synonymous					
MT-Cyb	14766	C>T	Missense	76.712%	T7I	T/S/T/S	Neutral (score: 0.48)	0.999
	15326	A>G	Missense	98.654%	T194A	T/M/I/I	Neutral (score: 0.4)	0.999
	16129	G>A	Intergenic					
	16213	G>A						
	16223	C>T						
	16290	C>T						
	16319	G>A						

# *Certified pathogenic mutation showed in bold*.

* *Conservation of amino acid for polypeptides in human(H), bovine(B), mouse(M) and xenopus lavis(X)*.

**Table 2:** The mtDNA variants in the severe patient (I'-2).

**Gene**	**Position^**#**^**	**Replacement (base)^**#**^**	**Gene region^**#**^**	**Mitomap** ** Frequency**	**Replacement** ** (amino)^**#**^**	Conservation (H/B/M/X)^*^	**Pathogenicity prediction** ** (APOGEE)**	**Reference**
	73	A>G	Intergenic					
	150	C>T						
	263	A>G						
	310	T>T/C						
	489	T>C						
12S rRNA	709	G>A	Non-coding					
	750	A>G						
	1019	A>G						
	1438	A>G						
16S rRNA	2706	A>G	Non-coding					
	3106	CN>C						
MT-ND1	3579	A>G	Synonymous					
MT-ND2	4769	A>G	Synonymous					
	4833	A>G	Missense	1.031%	T122A	T/I/I/L	Neutral (score: 0.35)	0.999
	5108	T>C	Synonymous					
MT-CO1	7028	C>T	Synonymous					
MT-CO2	7867	C>T	Synonymous					
	8200	T>C	Synonymous					
MT-ATP6	8643	C>T	Synonymous					
	8701	A>G	Missense	32.509%	T59A	T/S/L/Q	Neutral (score: 0.29)	0.999
	8860	A>G	Missense	98.503%	T112A	T/A/A/T	Neutral (score: 0.27)	0.992
MT-CO3	9540	T>C	Synonymous					
MT-ND3	10398	A>G	Missense	43.466%	T114A	T/T/T/A	Neutral (score: 0.44)	0.999
	10400	C>T	Synonymous					
MT-ND4	10873	T>C	Synonymous					
	11719	G>A	Synonymous					
	**11778**	**G>A**	**Missense**	**0.341%**	**R340H**	**R/R/R/R**	**Pathogenic (score: 0.97)**	0.999
MT-ND5	12705	C>T	Synonymous					
MT-ND6	14569	G>A	Synonymous					
MT-Cyb	14766	C>T	Missense	76.712%	T7I	T/S/T/S	Neutral (score: 0.48)	0.999
	14783	T>C	Synonymous					
	15043	G>A	Synonymous					
	15301	G>A	Synonymous					
	15323	G>A	Missense	0.456%	A193T	A/A/A/S	Neutral (score: 0.49)	0.999
	15326	A>G	Missense	98.654%	T194A	T/M/I/I	Neutral (score: 0.4)	0.999
	15497	G>A	Missense	0.520%	G251S	G/G/G/G	Neutral (score: 0.47)	0.999
	15860	A>G	Missense	0.141%	I372V	I/I/I/V	Neutral (score: 0.34)	0.999
tRNA^Pro^	15968	T>C	Missense	0.460%		T/T/A/G		
	16223	C>T	Intergenic					
	16519	T>C						

# *Certified pathogenic mutation showed in bold*.

* *Conservation of amino acid for polypeptides in human(H), bovine(B), mouse(M) and xenopus lavis(X)*.

### Multiple mtDNA Variants Might Be Associated With Different Level of Mitochondrial Damage

**Figure 3:**
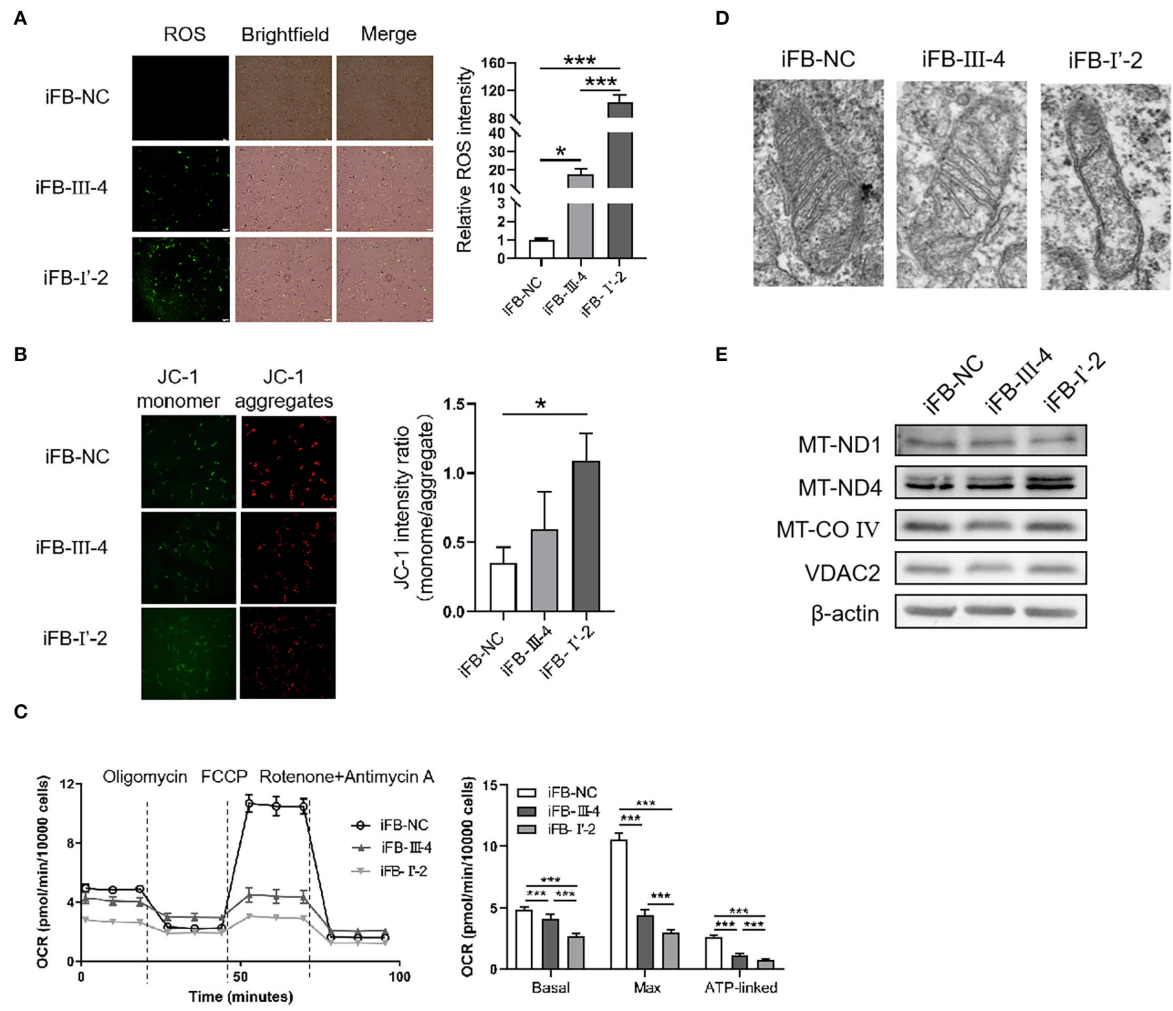
Mitochondria damaged severely in immortal fibroblast (iFB)-LHON cells with more variants. **(A)** ROS level was detection in different fibroblast cell lines: iFB-NC, iFB-III-4, and iFB-I'-2. *p ≤ 0.05, ***p ≤ 0.001. **(B)** JC-1 monome and aggregate were pictured. The ratio of fluorescence intensity between monomer and aggregate characterized mitochondrial membrane potential. *p ≤ 0.05. **(C)** oxygen consumption rate (OCR) value of three fibroblast cells was detected using seahorse. The basal (OCR value = OCR value at the beginning, meaning the level of energy metabolism rate in basal state), max (OCR value = OCR value at the highest point after FCCP treatment, meaning the maximum respiration rate that the cell can achieve) and ATP-linked (OCR value = OCR value decreased after oligomycin treatment, meaning the respiration part used for ATP synthesis) OCR values were calculated. ***p ≤ 0.001. **(D)** Mitochondrial morphology in iFB-NC, iFB-III-4, and iFB-I'-2 cells was photographed by electron microscope. **(E)** The protein levels of MT-ND1, MT-ND4, MT-CO IV, and VDAC2 were detected by Western blot. The β-actin was used as reference.

To investigate whether the number of variants in mtDNA affected mitochondrial function, we obtained skin tissue from the two probands and constructed immortal fibroblast cell lines (iFB-LHON: iFB-I'-2 and iFB-III-4). Fibroblast cells derived from the skin of subject without known mtDNA or nuclear gene mutations were used as controls (iFB-NC). The binocular visual acuity of control subject was 1.0 and no abnormality was found in visual field examination and OCT fundus imaging. First, ROS levels were detected in iFB cell lines. Fluorescence images showed that the ROS positive cells of iFB-LHON were much more than that of iFB-NC cells. Meanwhile, compared with iFB-III-4, iFB-I'-2 cells had higher ROS levels (Figure 3A). Mitochondrial membrane potential is an important indicator of mitochondrial function. The JC-1 test is an effective method for detecting mitochondrial membrane potential. When the potential was low, JC-1 mainly existed as a monomer, and the green fluorescence was enhanced; otherwise, the red signal was prominent. Therefore, the ratio of green-to-red fluorescence was used as a measure of the mitochondrial membrane potential. The results of the JC-1 test showed a slight decrease in the mitochondrial membrane potential in iFB-III-cells than that in iFB-NC cells, but the difference was no significant. However, the potential of iFB-I'-2 was significantly lower than that of iFB-NC and iFB-III-4 cells (Figure 3B). To further clarify mitochondrial function, we measured the oxygen consumption rate (OCR) of the iFB cells. The results showed that the basal and maximum OCR values of iFB-LHON cells were lower than those of iFB-NC cells. Among the two iFB-LHON cell lines, iFB-I'-2 had the lowest OCR in the basal, max and ATP-linked (Figure 3C). The results indicated that mitochondrial function was abnormal in iFB-LHON cells, and iFB-I'-2 had a more severe mitochondrial phenotype than that in iFB-III-4 cells, suggesting that multiple variations in mtDNA might be associated with the difference in mitochondrial function seen in these cell lines.

Electron microscopy was used to test whether multiple variants in mtDNA are associated with changes in mitochondrial morphology. The number of mitochondrial cristae was reduced in iFB-LHON cells, particularly in the iFB-I'-2 cell line (Figure 3D), indicating that multiple variations may be associated with morphological changes. However, we observed upregulated protein levels of MT-ND4 in iFB-I'-2 cells using Western blotting (Figure 3E). This might be due to the negative feedback caused by the deficiency of normal MT-ND4. However, the levels of other mitochondrial proteins such as MT-ND1, MT-CO IV, and VDAC2 were similar. These data suggested that the effect of multiple variants on mitochondrial function might be through the influence of the function of mitochondrial proteins rather than the expression level.

### Affected Energy Synthesis in Cells With Multiple Mitochondrial Variations

**Figure 4:**
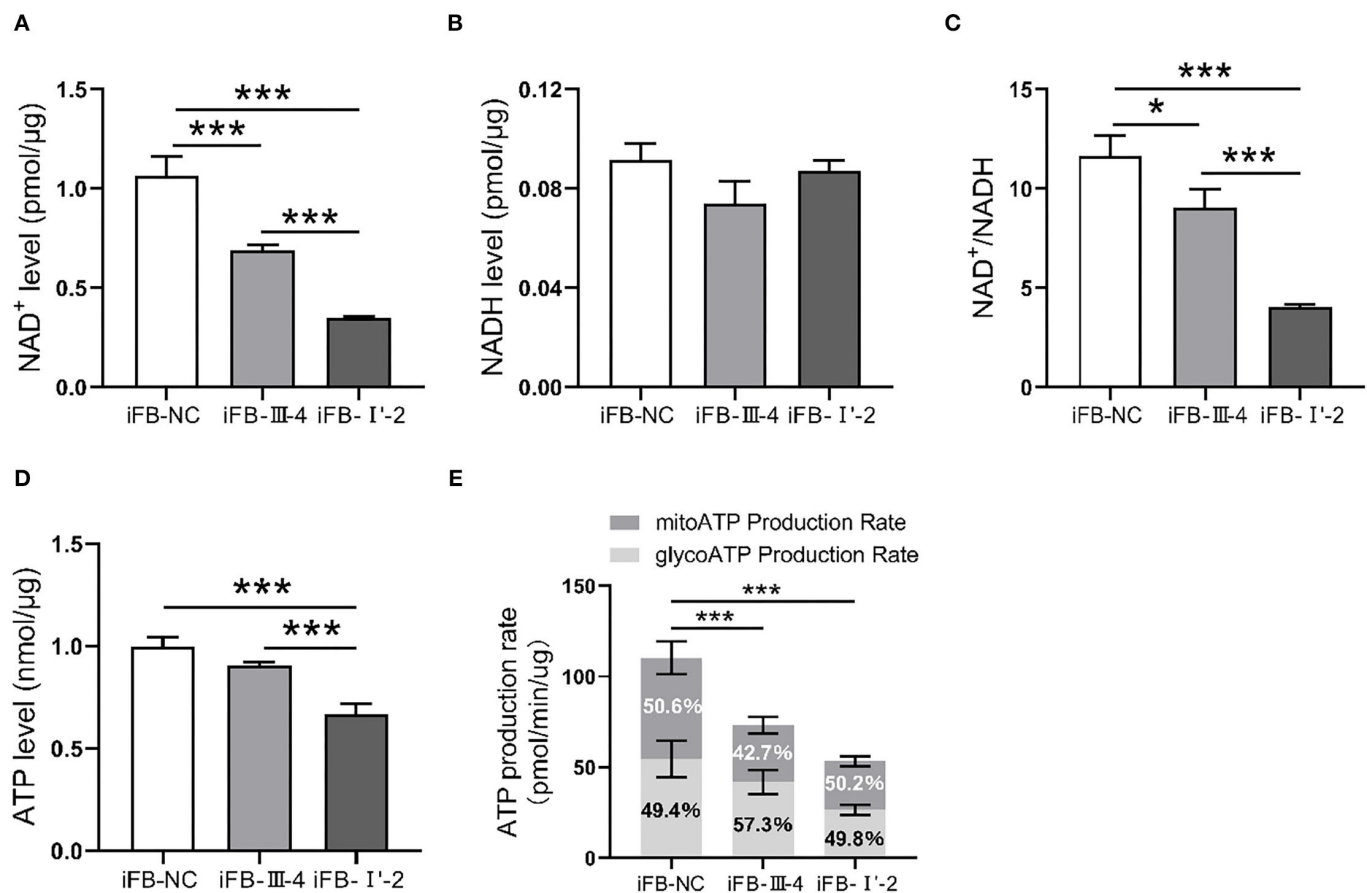
The iFB-LHON cells with more variants have lower energy synthesis capacity. **(A,B)** NAD^+^ and NADH level in mitochondria were tested in fibroblast cells. ***p ≤ 0.001. **(C)** The ratio of NAD^+^/NADH was calculated. *p ≤ 0.05, ***p ≤ 0.001. **(D)** Fibroblast cells were collected and ATP level in mitochondria was examined in both cells. ***p ≤ 0.001. **(E)** ATP production rates in fibroblast cells were detected by seahorse XF96 analysis. The mitoATP production rate refers to the ATP production rate related to mitochondrial oxidative phosphorylation (expressed as pmol ATP/min). The glycoATP production rate associated with the conversion of glucose to lactic acid in the glycolysis pathway (also expressed as pmol ATP/min). ***p ≤ 0.001.

Mitochondria are the most important organelles involved in cellular energy synthesis (Moos et al., 2021). Impaired function of the respiratory chain obstructs electron transfer and affects the synthesis of energy molecules. To determine whether multiple mtDNA variants affected energy synthesis, we measured the levels of NAD^+^, NADH, and ATP in mitochondria of these cell lines. NAD^+^ levels were lower in iFB-LHON cells than in iFB-NC cells, and iFB-I'-2 cells had the lowest levels of NAD^+^ (Figure 4A). However, there was no difference in NADH levels (Figure 4B). Therefore, the NAD^+^/NADH ratio was downregulated in iFB-LHON cells, particularly in iFB-I'-2 cells (Figure 4C). In addition, ATP levels showed the same trend as that of NAD^+^, and the lowest level was observed in iFB-I'-2 (Figure 4D). We further determined the ATP production rate in iFB cells. iFB-NC cells presented the highest ATP production rate, followed by iFB-III-4 and iFB-I'-2 cells which had the lowest rate (Figure 4E), suggesting that mtDNA variations might cause variations energy shortages.

### Multiple mtDNA Nucleotide Variants Resulted in Different Vulnerability to a Stress Stimulus

**Figure 5:**
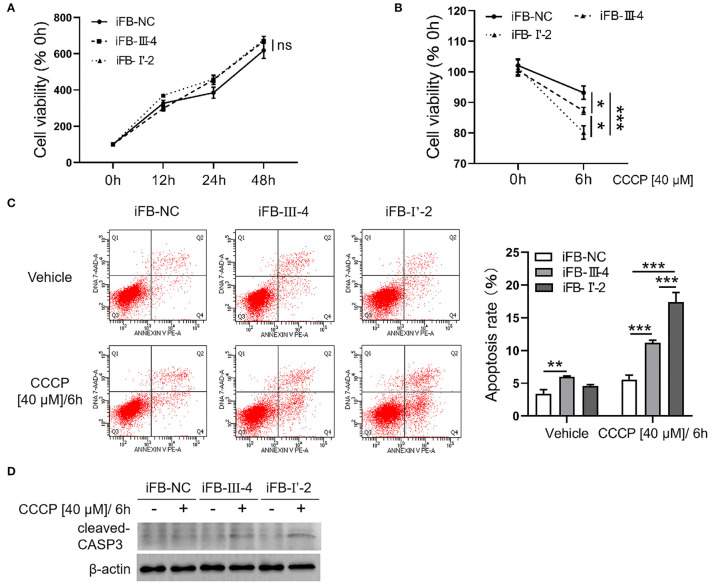
Fibroblast cells with more mtDNA variants were more sensitive to stimuli. **(A)** Fibroblast cells were cultured for 48 h. The cell viability was detected in 0, 12, 24, and 48 h, respectively, using CCK8. **(B)** Cells were treated with CCCP [40 μM] for 6 h and cell viability was measured before and after treatment. *p ≤ 0.05, ***p ≤ 0.001. **(C)** CCCP [40 μM/6 h] treatment was performed in fibroblast cells and apoptosis level was detected using flow cytometry. **p ≤ 0.01, ***p ≤ 0.001. **(D)** Fibroblasts cells were collected after CCCP [40 μM/6 h] treatment and cleaved-CASP3 was detected using western blotting.

Mitochondria are not only the energy center of cells but are also closely related to cell activities such as apoptosis and autophagy (Agarwal and Muqit, 2021; He et al., 2022). Therefore, we explored whether multiple mtDNA variants affect cell viability. The CCK8 assay showed that the cell viabilities of the three immortalized fibroblast (iFB) cells were similar (Figure 5A). The results suggest that in the fibroblast cells, energy deficiency associated with mtDNA variants had little effect on cell survival and proliferation. Mitochondria are sensors that respond to external stimuli. Stress responses can be triggered by mitochondrial damage or malfunction, resulting in apoptosis or cell death. Therefore, CCCP treatment was used to simulate the mitochondrial distress caused by external factors. Subsequently, the cell viability was measured at 0 and 6 h to evaluate cell survival. The cell survival was downregulated after the stimuli, though the sensitivity of the three cell lines was different. The survival rate of iFB-I'-2 and iFB-III-4 cells decreased to 80 and 87%, respectively, while the control cell line iFB-NC was the least affected and decreased to 92% (Figure 5B). Apoptosis was detected using flow cytometry. The apoptosis rate of iFB-I'-2 cells reached 17% after stimulation. The percentages of iFB-III-4 and iFB-NC cells were 11 and 6%, respectively (Figure 5C). The Western blotting was performed to detected cleaved caspase 3 after CCCP treatment. The results showed that the cleavage of caspase 3 in iFB-LHON cells was significantly enhanced after the CCCP stimulation and the cleavage band in iFB-I'-2 cells was the most significant (Figure 5D). These data imply that cell vulnerability to external stimuli varies among multiple mtDNA variants.

## Discussion

Phenotype heterogeneity is typical in LHON and is also a factor influencing the diagnosis and treatment of the disease (Jha et al., 2021). The reason for this heterogeneity is currently unclear and may be related to a variety of factors, such as the environment, epigenetic regulation, and nuclear gene modification. Recent studies have shown that polygenic mutations have an important impact on the occurrence and development of LHON. The number of different gene mutations may affect phenotype severity and disease progression, which may also causes heterogeneity (Jiang et al., 2016; Ji et al., 2020; Yu et al., 2020). Research on the relationship between this factor and heterogeneity cannot only extend the understanding of pathogenesis but also provide an important reference for the diagnosis and prognosis of LHON.

The two probands included in the study, both male and of similar age, were diagnosed with LHON based on their clinical symptoms and gene sequencing results (both had *MT-ND4* 11778G>A mutation). When the patients first visited the hospital, their clinical manifestations were similar. However, in a 2-year follow up, the phenotype of the patients varied. The visual acuity of patient III-4 was stable, while that of patients I'-2 declined progressively. Fundus photographs and SS-OCT results further confirmed diseases heterogeneity.

Most LHON pathogenic mutations are in mitochondrial genes that encode proteins as respiratory chain subunits. With potential additional protein modifications, the multi-subunit mutations may cause more severe respiratory chain damage than single-subunit mutations (Ji et al., 2020). Therefore, mtDNA variants may cause different degrees of mitochondrial damage, resulting in inconsistent phenotypes. Sequencing results showed that, in addition to the common 11778G>A mutation, the two patients also showed variations in other mitochondrial gene. However, these variants were not proven to be pathogenic when they existed individually. Patient III-4 contained six missense variants, whereas patient I'-2 contained 11 missense variants. The conservative analysis showed that more conservative amino acids were present among the variants in patient I'-2 than in patient III-4. Although these variants have not been defined as pathogenic, multi-locus variations may exacerbate mitochondrial damage in a cumulative manner, thereby contributing to disease progression.

The mutant genes in the two patients were mainly concentrated in the coding region of *MT-ND2, MT-ND3, MT-ND4, MT-ATP6*, and *MT-cyb*, but the numbers were different. Therefore, the respiratory chain function of the patient's cells might be different. We thus constructed a skin fibroblast cell line (iFB cells) and evaluated the respiratory function of the cells by measuring the OCR values. The results showed that the basic OCR value of the cells was significantly lower than that of the control cells. Among the two patients, patient I'-2 had a lower OCR value, indicating that the respiratory capacity was more damaged than that of the mild patient. ATP-linked respiration reflects the ATP synthesis ability of cells, and also showed the lowest value in patient I'-2. This trend corresponded to the ATP levels in iFB cells. Subsequently, the maximum respiratory rate was generated by CCCP stimulation. Here, the OCR value showed a significant difference, indicating insufficiency of the patients' respiratory potential and abnormal respiratory chain function. The ROS level in the iFB-I'-2 cells was found to be higher, and the mitochondrial membrane potential decreased more significantly. Mitochondrial damage in patient I'-2 was severe.

In addition to their involvement in energy synthesis, mitochondria also play an important regulatory role in cell survival and apoptosis. The CCK8 test results showed that iFB-LHON and iFB-NC cells had similar viability and proliferation rates. However, when cells were treated with CCCP to simulate harmful external stimuli, the patients' iFB cells were more vulnerable to injury and iFB-I'-2 cells were the most affected. The results indicated that although mitochondrial dysfunction had little effect on the survival and proliferation of patient fibroblasts under normal conditions, iFB-LHON cells were more susceptible to stress. It is possible that the responses to stimuli in cells bearing more mtDNA variants were exaggerated.

Due to the inability to obtain retinal tissue from patients, skin fibroblasts have recently been used as models for molecular studies of genetic eye diseases (Morvan and Demidem, 2018; Uittenbogaard et al., 2019; Kishita et al., 2021). The cell lines had the same mutations as the other somatic cells, were easy to obtain, and were consistent with the patient's genetic background. We prove that the mitochondria function of iFB-LHON cells was abnormal and that these cells were more prone to apoptosis than control cell lines. These data suggest that skin fibroblast cell lines could reflect some cellular phenotypes of RGC cells in patients with LHON. Because of lower oxygen consumption than retinal ganglion cells and photoreceptors (Almasieh et al., 2017; Chen et al., 2019), skin-derived cells might be more resistant to hypoxemia, and could still be used as alternative models for LHON research. Inevitably, some limitations existed in the current study. The data presented are based on a limited number of patients and a bigger cohort is necessary to verify the conclusions in the future. In addition, changes in mitochondrial membrane potential should be further validated using TMRE (tetramethylrhodamine, ethyl ester) as it is more stable and efficient (Perry et al., 2011).

By studying mitochondrial function, we found that iFB-LHON cells with multiple mtDNA variants had weaker respiratory function, more severe mitochondrial damage, and lower levels of energy molecules such as NAD^+^ and ATP. Although a variant alone is non-pathogenic, additional multiple mutations might affect protein functions by regulating its conformation, consequently leading to mitochondrial dysfunction (Zhu et al., 2016; Guo et al., 2017; Agip et al., 2018). Different levels of mitochondrial damage resulted in different phenotypes, and caused heterogeneity. Exaggerated damage by more mutations has been reported in another blood-derived cell line. Guan et al. found that immortalized lymphocytes containing two pathogenic LHON mutations had more severe mitochondrial damage than cells with a single mutation (Ji et al., 2020). Several factors contribute to the severity of LHON. Although the position of the mutation is more important, previous, and current studies suggest that more mtDNA variants may deteriorate mitochondrial damage and contribute to the different phenotypes of LHON.

## Materials and Methods

### Patients and Clinical Assessment

This study was performed in accordance with the Declaration of Helsinki and approved by the Ethics Committee of Henan Eye Hospital. Patients and healthy subjects were recruited from Henan Eye Hospital. All participants signed informed consents forms after a detailed explanation of their risks and benefits. All patients and their direct relatives underwent a complete ophthalmic examination, such as best-corrected visual acuity (BCVA), color vision, visual field, slit-lamp biomicroscopy, intraocular pressure (IOP), and SS-OCT.

### Gene Sequencing and Analysis

Genomic DNA was isolated from peripheral blood of all subjects with the TIANGEN Blood DNA Kit (DP304, TIANGEN, Beijing, China). Nanodrop 2000 was used to detect the DNA concentrations. Concentrations above 25 μg/μl and OD260/280 between 1.8 and 2.0 were used for the next steps. Sequencing and alignment were performed on the Nextseq 500 (Illumina, San Diego, CA, USA) platform. Filter the original read to delete low quality reads (Q30 ≥ 80% was defined as qualified). Clean data were mapped using BWA (Burrows-Wheeler Alignment tool). The mtDNA was sequenced with a depth greater than X5000. The Sanger sequencing was conducted to verify disease-relevant mutations.

### Construction of Immortalized Fibroblasts Cells

Human fibroblast cells were isolated from 2 mm^2^ dermal punch biopsies. Cells were transfected with the SV40 virus to prepare iFB cells. iFB cells were cultured in a complete fibroblast medium (Lab050-NP, Kuisai, Shanghai, China). The cells were generated with informed consent and ethics approval [HNEECKY-2019(12)].

### ROS Measurement

Fibroblasts were seeded in a 6-well plate and cultured overnight. DCFH-DA (S0033S, Beyotime, Shanghai, China) was diluted in serum-free medium at a ratio of 1:1000. Diluted DCFH-DA was added to the wells and cultured in a 37°C incubator for 40 min. Fluorescent images were captured using a microscope after washing the cells thrice with serum-free medium.

### Mitochondrial Membrane Potential Assay

The mitochondrial membrane potential assay was performed using a JC-1 detection kit (C2006, Beyotime). The cells were incubated overnight in a 6-well plate. After washing the cells once with PBS, 1 ml JC-1 staining working solution and 1 ml serum-containing medium were added to each well. Cells were cultured at 37°C in an incubator for 20 min. After incubation, the supernatant was discarded and the cells were washed twice with JC-1 buffer. Finally, 2 ml of serum-containing medium was added to the cell wells, and green and red fluorescent photos were taken. The intensity ratio of green and red fluorescence represents the mitochondrial membrane potential.

### Determination of Oxygen Consumption Rate

The function of the respiratory chain is reflected in the oxygen consumption rate (OCR value) of cells. The OCR values were measured using an XFe analyzer (XFe96, Agilent Seahorse Technologies, Palo Alto, CA, USA). Fibroblast cells were seeded in the seahorse plate (102601-100, Agilent Seahorse Technologies) at 12,000 cells per well and incubated overnight. The cell medium was replaced with an XF base medium (103334-100, Agilent Seahorse Technologies) containing 2 mM glutamine, 1 mM pyruvate, and 10 mM glucose; and the seahorse plate was placed in a 37°C incubator without CO_2_ for 1 h. Four inclusions from the XFe cell Mito stress test kit (103015-100, Agilent Seahorse Technologies) were diluted into the following concentrations and injected into the probe plate during detection: oligomycin (15 μM), p-trifluoromethoxy phenylhydrazone [20 μM], and a mixture of antimycin A (5 μM) and rotenone (5 μM). Cell numbers were calculated for normalization. The software Wave 2.6.3 was used for the analysis.

### Cell Viability Detection

The cells were cultured overnight in 96-well plate. The CCK8 (CK04, Dojindo, Rockville, MD, USA) was diluted with basal medium without serum at a ratio of 1:100 to prepare the CCK8 working solution. The CCK8 working solution (100 μl/well) was added and the cells were incubated for 3 h. The absorbance was then determined at 450 nm. The value at 0 h was taken as the origin to calculate the relative value at each time point.

### Apoptosis Assay

The apoptosis assay was performed following the instructions of the PE Annexin V Apoptosis Detection Kit I (559763, BD Biosciences, Franklin Lakes, NJ, USA). Briefly, the number of 1 × 10^5^ cells were collected after treatment with or without CCCP [40 μM] (HY-100941, Newark, NJ, USA) for 6 h. Cells were washed using cold PBS and resuspended in 100 μl 1× binding buffer. PE-annexin V (5 μl) and 7-AAD (5 μl) were added to the tube and samples were incubated for 15 min at room temperature in the dark after gentle vortexing. Samples were added to 400 μl 1× binding buffer for flow cytometry.

### ATP and NAD^+^ Detection

ATP and NAD^+^ were detected using an ATP assay kit (S0027, Beyotime) and a NAD^+^ assay kit (S0175, Beyotime) according to the manufacturer's instructions, and the protein concentration was used as criterion for normalization.

### Western Blot

Cells were lysed in RIPA lysis buffer (PC101, Epizyme Biomedical Technology, Shanghai, China) with 1X protease Inhibitor cocktail (GRF101, Epizyme Biomedical Technology) and incubated on ice for 30 min. After centrifugation at 12,000 rpm for 30 min, the supernatant was mixed using 5× loading buffer. Subsequently, the supernatants were boiled at 95°C for 5 min. The samples were electrophoresed using 12% SDS-PAGE (PG113, Epizyme Biomedical Technology) and transfected into PVDF membranes (IPVH00010, Millipore, USA). After blocking with 5% milk powder (PS112L, Epizyme Biomedical Technology) for 1 h, the membranes were incubated with primary antibodies overnight at 4°C: MT-ND1(385032, ZENBIO, Chengdu, China), MT-ND4(PA5-97298, Invitrogen, Carlsbad, CA, USA), MT-CO IV (11967s, Cell Signaling Technology, Danvers, Massachusetts, USA), VDAC2 (11663-1-AP, Proteintech, Wuhan, China), cleaved-CASP3 (ab2302, abcam, London, UK) and β-actin (200068-8F10, ZENBIO). After washing thrice, the membranes were incubated with secondary antibodies, goat anti-mouse IgG-HRP (abs20039, Absin, Beijing, China), and goat anti-rabbit IgG-HRP (abs20040, Absin) for 1.5 h at room temperature. Finally, the membranes were exposed using a chemiluminescence apparatus (GelView6000Plus, Guangzhou, China).

### Statistical Analysis

Image Pro Plus (National Institutes of Health, Bethesda, MD, USA) was used to analyze fluorescence intensity. GraphPad Prism 8.0 (GraphPad, La Jolla, CA, USA) was used for statistical analyses. Data in the graphs are shown as means ± standard deviations. One-way analysis of variance (ANOVA) followed by the Dunnett's *post hoc* test was used to evaluate the differences. The statistical significance was set at *p* ≤ 0.05.

## Conclusion

In conclusion, we found that in fibroblast cells derived from LHON patients baring G11778A mutation, multi-mtDNA variants might contribute to the variety of mitochondrial function and response to external stimulus. Thus, the multi-mtDNA variants may contribute to the heterogeneity of LHON.

## Data Availability Statement

The raw data supporting the conclusions of this article will be made available by the authors, without undue reservation.

## Ethics Statement

The studies involving human participants were reviewed and approved by Ethics Committee of Henan Eye Hospital [HNEECKY-2019(12)]. The patients/participants provided their written informed consent to participate in this study.

## Author Contributions

SY conducted experiments, data analysis, manuscript writing, and financial support. QZ, XJ, and LY conducted data analysis. YL and QG collected clinical data. MY constructed the fibroblast cells. FQ performed flow cytometry. BL conceptualized the design, provided the materials and the financial support, revised, and gave final approval for the manuscript. All authors contributed to the article and approved the submitted version.

## Funding

This research was funded by the Key Technologies Research and Development Program of Henan Science and Technology Bureau (212102311009), Natural Science Foundation of Henan Province (222300420195), and National Natural Science Foundation of China grants (82004001 and 82071008).

## Conflict of Interest

The authors declare that the research was conducted in the absence of any commercial or financial relationships that could be construed as a potential conflict of interest.

## Publisher's Note

All claims expressed in this article are solely those of the authors and do not necessarily represent those of their affiliated organizations, or those of the publisher, the editors and the reviewers. Any product that may be evaluated in this article, or claim that may be made by its manufacturer, is not guaranteed or endorsed by the publisher.
